# Effectiveness and compliance of an oscillating-rotating toothbrush in patients with dental implants: a randomized clinical trial

**DOI:** 10.1186/s40729-018-0150-6

**Published:** 2018-12-10

**Authors:** Giuseppe Allocca, Diana Pudylyk, Fabrizio Signorino, Giovanni Battista Grossi, Carlo Maiorana

**Affiliations:** 1 Center for Edentulism and Jaw Atrophies, Maxillofacial Surgery and Dentistry Unit, Fondazione IRCCS Cà Granda – Ospedale Maggiore Policlinico, University of Milan, Via Commenda 10, 20122 Milan, Italy; 2 Oral Surgery, Maxillofacial Surgery and Dentistry Unit, Fondazione IRCCS Cà Granda – Ospedale Maggiore Policlinico, University of Milan, Via Commenda 10, 20122 Milan, Italy

**Keywords:** Dental implant, Domiciliary hygiene, Electric toothbrush, Implant maintenance, Oral hygiene

## Abstract

**Background:**

The aim of this randomized clinical trial was to assess the efficacy of an oscillating-rotating toothbrush in reducing plaque and inflammation around dental implants.

**Methods:**

Eighty patients presenting dental implants were enrolled in this study and assigned randomly to two different groups: 40 patients in the test group and 40 in the control one. Each patient in the test group received an oscillating-rotating toothbrush while in the control group patients kept using the manual toothbrush. Furthermore, the test group received a special toothbrush head designed for dental implants and another one for natural teeth. Domiciliary oral hygiene instructions were given to both groups. Periodontal parameters like plaque index (PI), bleeding on probing (BoP), and probing pocket depth (PPD) were recorded at the baseline and after 1 and 3 months.

**Results:**

At the end of the study, the difference of plaque and bleeding indices with the baseline was statistically significant for both test and control groups (*P* < 0.0001). Implant sites showed higher values of both BoP and PI when compared to the natural teeth. In the second part of the study, comparing the 1–3-month period, the oscillating-rotating toothbrush was effective in reducing new plaque formation (*P* < 0.0001) and bleeding (*P* < 0.0001) both at the implant sites and the dental sites comparing to manual ones (*P* > 0.05). No significant differences were appreciated concerning the PPD.

**Conclusions:**

The oscillating-rotating toothbrush can be successfully used for the plaque and bleeding control of the peri-implant tissues.

## Background

Dental implants became one of the most accepted treatments for the rehabilitation of partial or complete edentulism [1]. However, inflammatory processes may still occur due to the presence of the implant itself [2]. It is well known that peri-mucositis and peri-implantitis are strictly related to the presence of plaque on the surface of the implant-prosthetic complex, which lead respectively to the inflammation of peri-implant soft tissues and the bone loss around the implant neck area [3, 4]. The problem of implant maintenance must be taken in serious consideration even before the dental implant placement. Many risk factors have been associated to peri-implantitis such as smoke, diabetes, and a history of periodontal disease [5–8]. Furthermore, the prevalence of this pathology is rising. It has been estimated, in fact, that a range from 10 to 43% of all implants placed today will have some form of peri-implantitis in about 10 years [9, 10]. Many authors associated the microbiological flora responsible of peri-implantitis to the one associated to periodontal disease, while others confuted this hypothesis [11].

Many techniques and protocols have been introduced for the treatment of peri-implantitis; however, the topic is still debated and the different rates of success of various treatments still suggest that a good prevention must still be preferred [12]. The presence of bacterial microfilm on the implant surface has been individuated as the primary cause of the pathologic mechanism. As well as in the teeth, mechanical removal represents the only treatment able to remove the microfilm and toothbrush and dental floss are the only effective domiciliary devices able to remove plaque from the teeth and dental implant. Mouth rinses or other methods may enhance periodontal indices but only when associated to an effective primary mechanic removal device. It has also been proved how both manual and electric toothbrushes are effective in the plaque removal [13]. Several authors comparing the two devices were not able to find any differences in term of clinical results, while others found advantages for one technique with respect to the other [14–17]. Patients with motor problems and elderly may found benefit in using the electric toothbrush, which does not require the same level of manual skills as the manual one [18, 19]. Recently, there has been introduced a new type of electric toothbrush, with a visual-sound system, showing the correct pressure to apply when brushing and the exact amount of time necessary to complete one or half dental arch. Special designed toothbrush heads for different areas of the mouth and different surfaces, like dental implants, have recently been introduced for electric toothbrushes without a clear scientific support. The present study aims to investigate the efficacy of an oscillating-rotating toothbrush using a dedicated designed head, in patients with dental implants.

## Methods

**Fig. 1:**
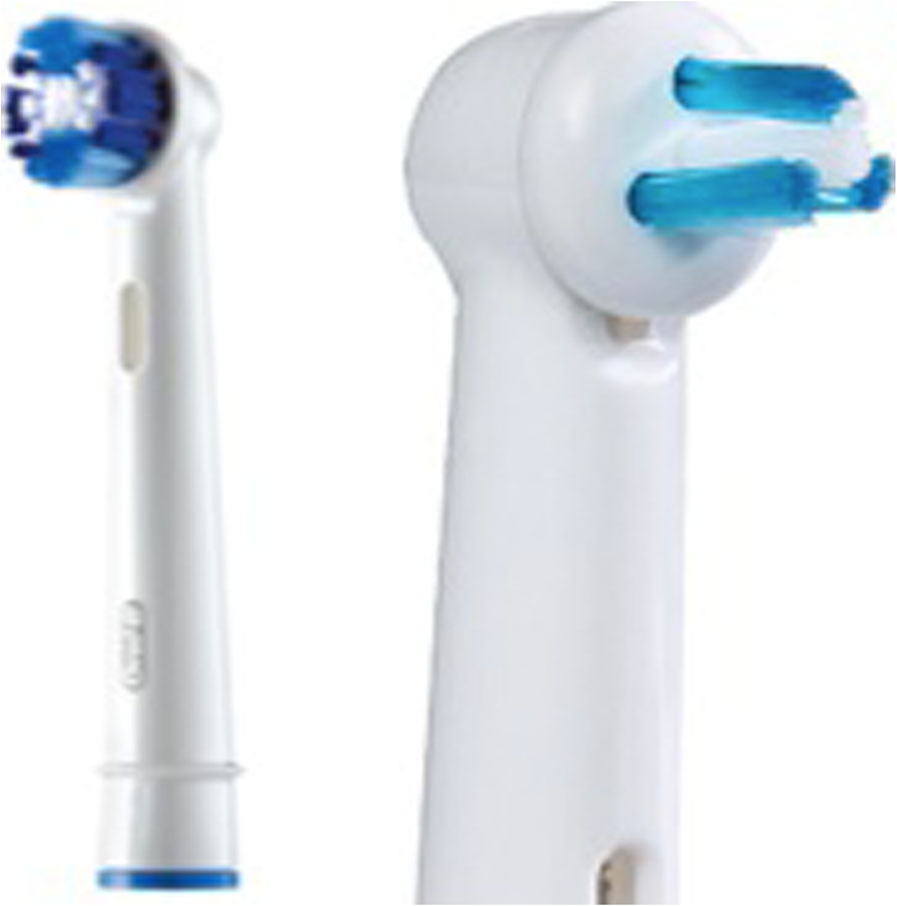
Electric toothbrush heads: on the left is the one designed for natural teeth, and on the right is the one designed for dental implants

The study was conducted between September 2015 and June 2017 at Implantology Department of Policlinic Hospital, University of Milan, Milan. It was designed as a monocentric randomized clinical study according to the STROBE criteria. Eighty patients who underwent dental implant rehabilitation were selected for this study. At the screening visit, subjects were asked to read and sign a written informed consent and personal medical history and demographic information was obtained. Dental implants must have been placed at least 1 year before the recruitment; other inclusion criteria were age between 18 and 90 and a good general health. Patients with orthodontic therapy or removable prosthesis, including overdenture type, were not included in the study as well as non-controlled diabetic or heavy smoker (> 10 cigarettes) patients. The patients were already following a maintenance program after the implant placement; however, all of them were using the manual toothbrush for domiciliary oral hygiene. After being included in the study, each patient underwent periodontal (North Carolina) and peri-implant (perio probe) charting and recording of bleeding and plaque indexes (gingival bleeding index and plaque control record). Gingival bleeding index and plaque control record were recoded as the presence/absence of bleeding or plaque on four sites per tooth/implant. In order to detect the plaque, a disclosing agent was used. Sequentially, dental hygienist performed professional prophylaxis to establish a plaque free dentition. A software program randomly assigned 40 patients for both test and control groups. The electric toothbrush (Oral-B® ProfessionalCare 6000 with Bluetooth; Oral-B®, Procter & Gamble, Cincinnati, OH, United States) was introduced to patients of the test group, and instructions were given. According to the producer instruction, the procedure must have lasted not less than 2 min, using a timer set on 30 s for quadrant, twice/day. Furthermore, all the patients received a special toothbrush head designed for dental implants (Interspace; Oral-B®) together with another one for the natural teeth (Precision clean; Oral-B®) (Fig. 1). The patients of the control group did not change the manual toothbrush as a domiciliary oral hygiene device and received instructions of the modified Bass technique. The recommended time for toothbrushing was at least 90 s, twice a day. Patients of both groups received all the information in a paper copy. Once verified that the patients understood the instructions, new appointments were scheduled after 1 and 3 months. Bleeding on probing, plaque index, and probing depth were recorded at each visit on both dental implants and natural teeth. The entire sample had to use the same toothpaste to reduce the variability of the results.

### Statistical analysis

Mean scores of all clinical indices for each subject were calculated separately for dental implants and natural teeth. The final data analysis was performed for those subjects who completed the study. The Student’s *t* test and the Mann-Whitney *U* test were used to evaluate whether any statistically significant differences were present between the two groups at each time point, and the Wilcoxon signed-rank test was performed to verify if any statistically significant changes occurred from baseline within each group. A total sample size of 74 patients (37 per group) achieves 81% power to detect a difference of 0.2 between the differences of group means with group standard deviations of 0.3. *P* values < 0.05 were considered statistically significant.

## Results

**Fig. 2:**
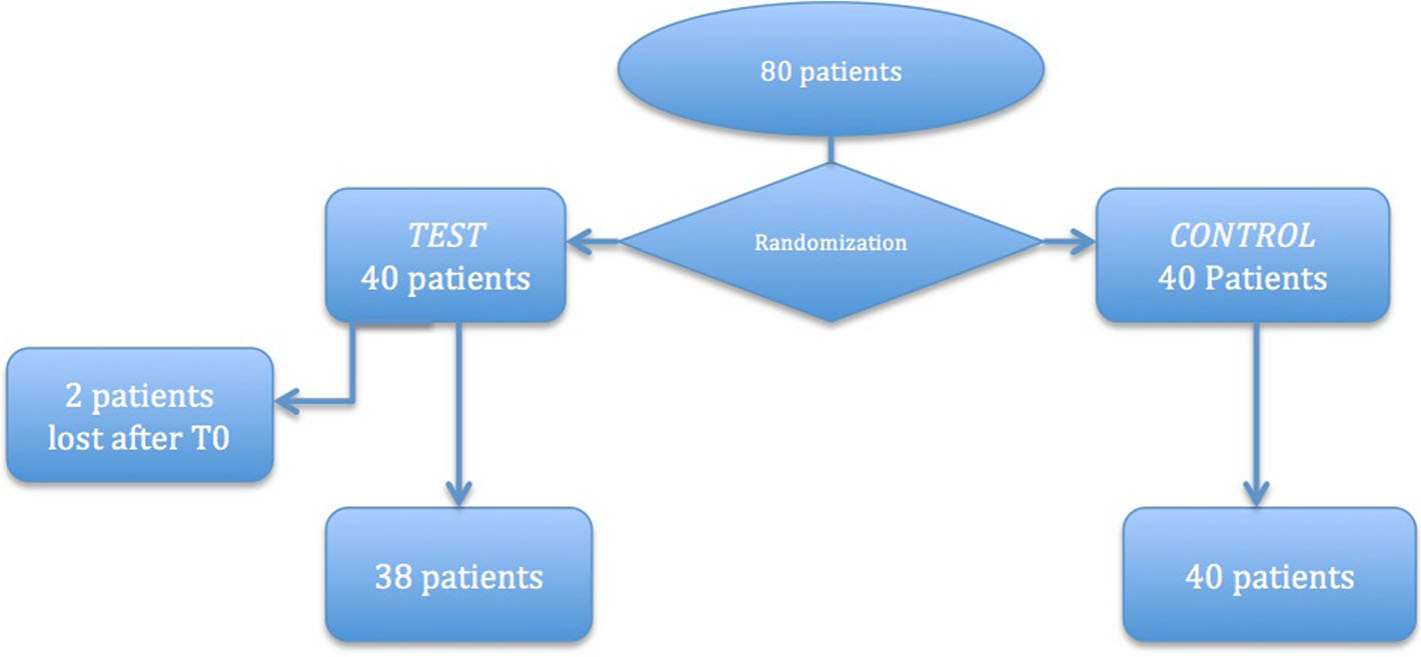
Patients’ population flow chart

Seventy-eight patients successfully completed the study (45 women and 33 men aged from 31 to 76 years old) (Fig. 2). Two patients of test group did not show up both at the first and second controls. No patients were excluded or showed complications or adverse reaction. Results are shown in Table 1. The average number of implants per patients was 4.8 ± 3.4 in the control group and 4.4 ± 2.9 in the test one. Single crowns, implant-supported bridges, and Toronto bridge were included in both of the study groups. The values taken in consideration were recorded for both the dental implants and the rest of the dentition and compared at each time. All dental implant index values were higher when compared to the natural teeth ones while no differences were appreciable concerning the PPD. The study provided data for the test and control groups at three different time points. Analyzing the results, it can be observed for both groups a high decrease of BoP and PI values after 1 month after the baseline, related to the prophylaxis performed by dental hygienist.

**Table 1:** BoP, PI, and PPD mean values at baseline, 1 month, and 3 months

	Baseline	1 month	3 months
	T0	T1	T2
BoP implants, test	46.55% ± 18.41%	32.31% ± 13.27%	22.18% ± 11.06%
BoP implants, control	32% ± 24.88%	19.84% ± 15.52%	19.11% ± 17.30%
BoP teeth, test	18.81% ± 15.93%	8.76% ± 8.11%	6.5% ± 5.18%
BoP teeth, control	21.61% ± 15.38%	15.50% ± 12.21%	16.38% ± 11.79%
PI implants, test	53.71% ± 14.72%	33.65% ± 12.57%	15.52% ± 12.29%
PI implants, control	50.13% ± 27.39%	28.66% ± 16.26%	32.68% ± 16.02%
PI teeth, test	33.15% ± 13.49%	20.76% ± 10.16%	14.5% ± 6.74%
PI teeth, control	41.34% ± 17.20%	32.26% ± 15.02%	35.77% ± 15.80%
PPD implants, test	2.73 mm ± 0.59 mm	2.67 mm ± 0.5 mm	2.61 mm ± 0.54 mm
PPD implants, control	2.4 mm ± 0.97 mm	2.22 mm ± 0.57 mm	2.21 mm ± 0.66 mm
PPD teeth, test	1.7 mm ± 0.47 mm	1.69 mm ± 0.38 mm	1.69 mm ± 0.4 mm
PPD teeth, control	2.01 mm ± 0.67 mm	1.93 mm ± 0.58 mm	2.04 mm ± 0.52 mm

Data are shown as mean ± standard deviation

*test* electric toothbrush with the two different heads designed for dental implants and natural teeth, *control* manual toothbrush

The second part of the study described the re-colonization of dental implants and teeth surfaces: this was related to the proper use of oral hygiene devices, showing the effective difference of the manual and electric toothbrushes in preventing the new plaque formation and the consequent inflammation status.

### Plaque index

**Fig. 3:**
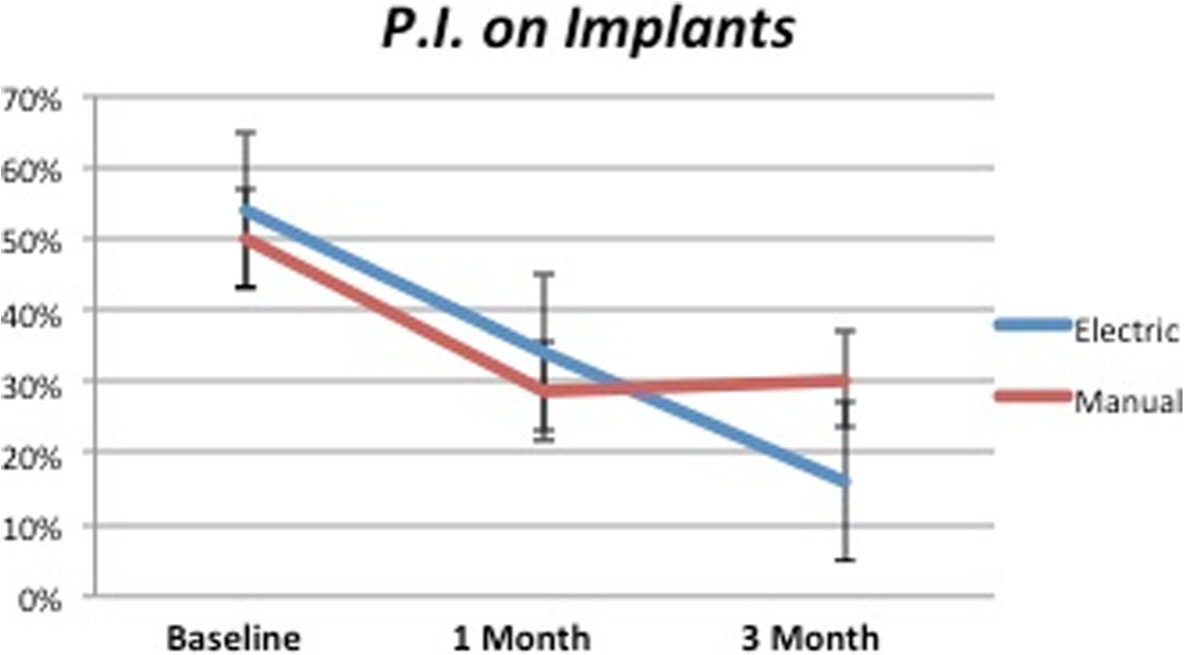
PI on dental implants. Test values keep reducing after 1 month while control maintains the same level

The difference of PI recorded around implants at the beginning and the end of the study was statistically significant for both control and test groups (*P* < 0.0001). Observing in detail the second part of the study in Fig. 3, it was possible to observe how the test group kept reducing (*P* < 0.0001) while the control showed a mild increase (*P* = 0.68). Comparison at 3 months showed statistical significance (*P* < 0.05).

**Fig. 4:**
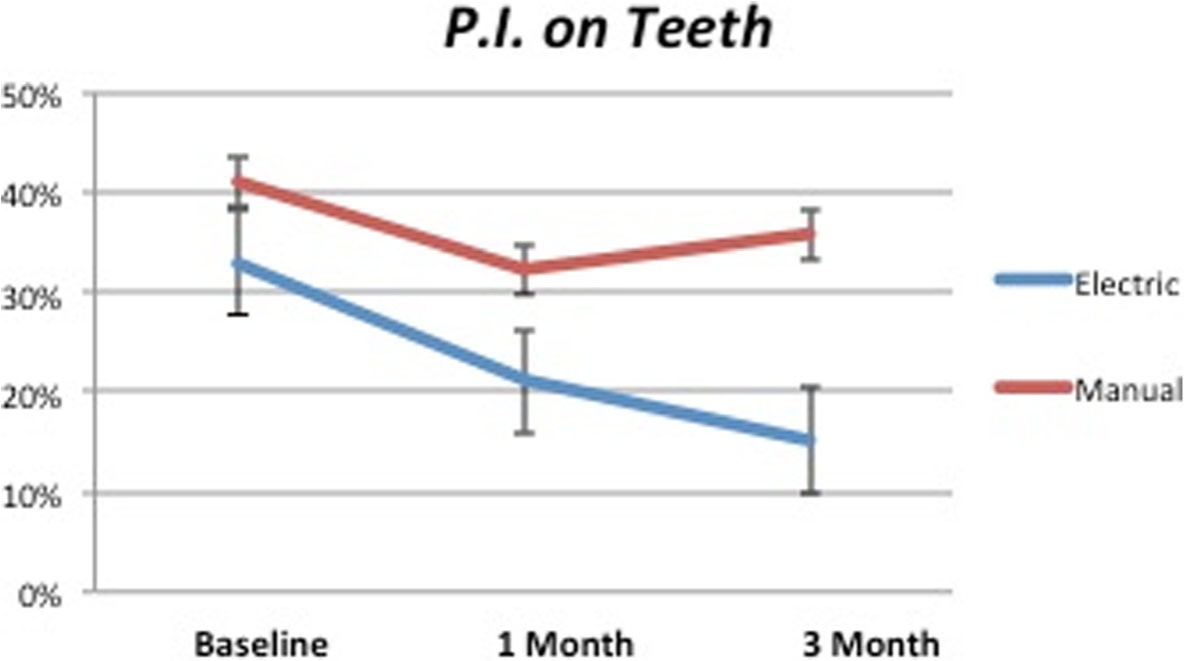
PI on natural teeth. After 1 month, the test group showed mild reduction while control a light improvement

In Fig. 4 is shown the PI recorded around natural teeth: in this case, comparing the baseline with the data collected after 3 months was possible to observe high statistical significance only for the test (*P* < 0.0001) and significance for the control (*P* < 0.05; *P* = 0.031). Highlighting the second part, the different trend of test and control lines confirmed the higher performances of test devices (*P* < 0.0001) compared to the control that showed a mild increase (*P* = 0.16). Comparison between the two groups at 3 months was highly statistically significant (*P* < 0.0001).

### Bleeding on probing

**Fig. 5:**
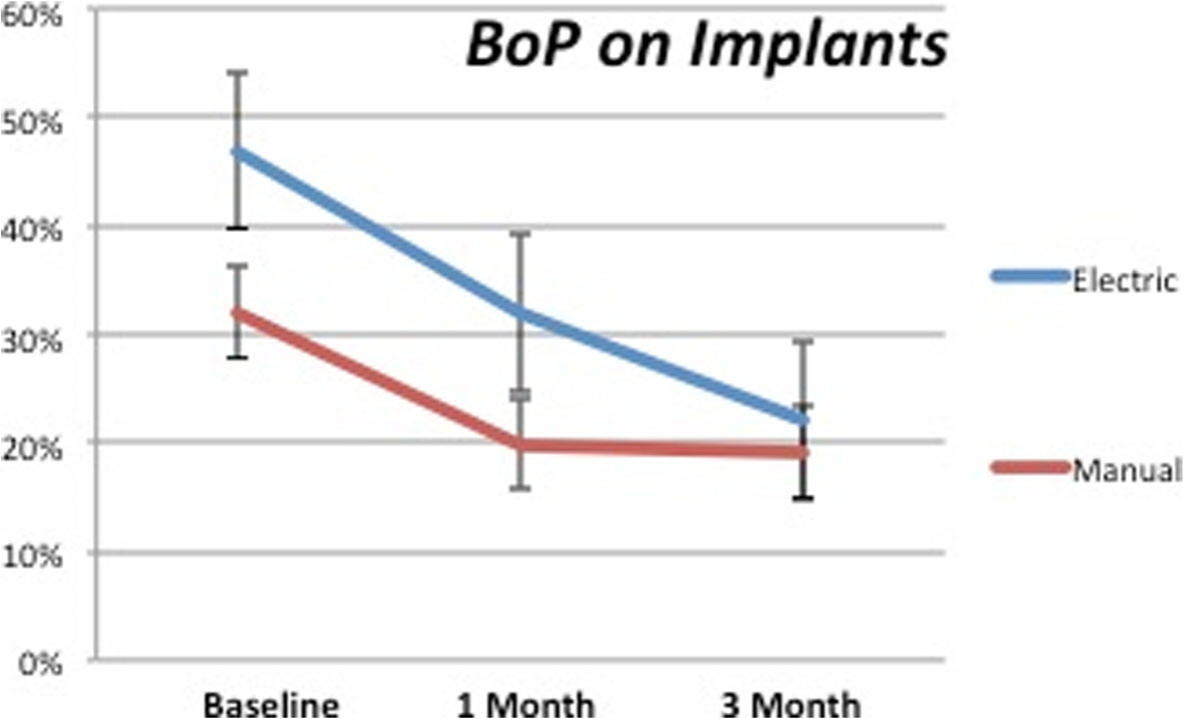
BoP on dental implants. It can be observed how the values keep decreasing after 1 month only in the test group

The difference between the BoP recorded on dental implant sites at baseline and the end of the study showed statistical significance for both the test and control groups (*P* < 0.0001) (Fig. 5). Analyzing in detail the 1–3-month period, it was observed how only the test group showed a statistical significance (*P* < 0.0001) while the control lost it (*P* = 0.709). At 3 months, no significative differences between the two groups were observed (*P* = 0.564).

**Fig. 6:**
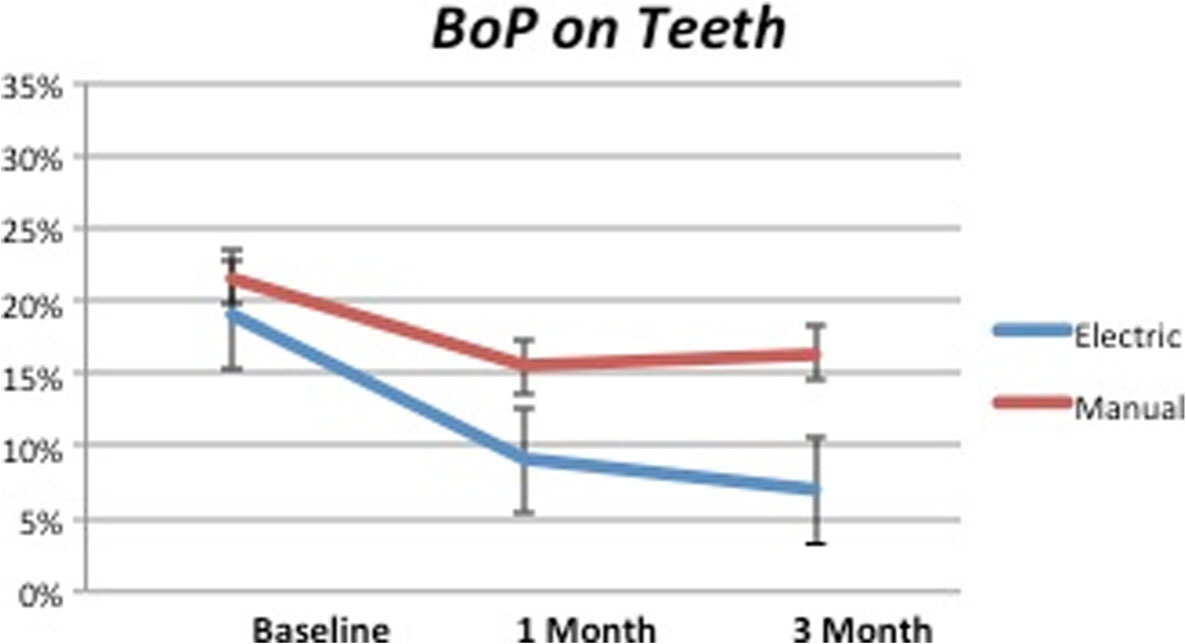
BoP on natural teeth. While the control group shows a mild increase between 1 month and 3 months, the test group values decrease during all the duration of the study

Analog situation could be observed in Fig. 6, representing BoP around the natural teeth. In this case, the difference with the baseline were significant for both groups (*P* < 0.0001 and *P* = 0.007 respectively for test and control). In the second part of the observation period (1–3-month period), it was possible to detect an increase for the control (*P* = 0.342) while the test kept decreasing, even if slightly (*P* < 0.05; *P* = 0.0021). The comparison between the test and control groups after 3 months showed a statistical significance (*P* < 0.05).

### Pocket probing depth

**Fig. 7:**
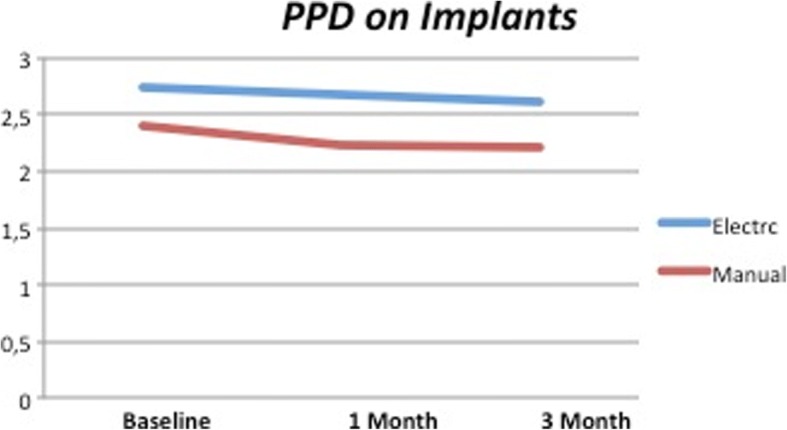
PPD on dental implants. No significant differences appreciable

**Fig. 8:**
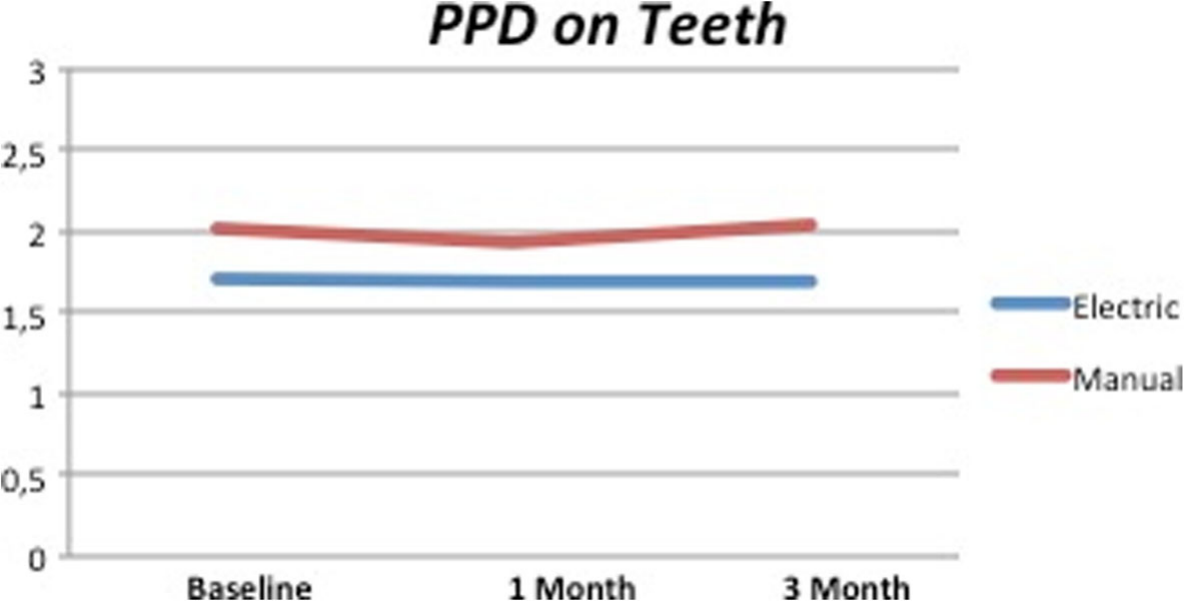
PPD on natural teeth. No significant differences appreciable

No differences during the time points were observed in both test and control groups as clearly shown in Figs. 7 and 8. It was possible to observe a reduction of PPD of 0.15 mm between the beginning and end of the study around dental implants on both test and control groups.

## Discussion

This 3-month study aimed to demonstrate the efficacy of an electric toothbrush in reducing plaque and gingival inflammation around dental implants and natural teeth. To better understand the different data collected around two different anatomical structures, we decided to collect data separately. Analyzing our results, it is possible to observe how the mean values for probing, bleeding, and plaque index were bigger for dental implants. According to literature, it was expected to find deeper probing for dental implants [20]. Many authors associated this to the different kind of attachment and the different orientation of periodontal fiber around dental implants [21–23]. The electric toothbrush has widely been described as a preventive option in the maintenance of peri-implant tissues [24–28]. However, many authors did not observe any differences between the manual and electric toothbrush, and for this reason, the topic is still controversial [17, 19]. In the present study, the manual toothbrush seemed to maintain the values achieved with the professional prophylaxes; however, a mild increase of both PI and BoP was detected after 3 months. The choice to perform prophylaxis on all patients after baseline index recording was done in order to bring the patients at the same level and reduce the variability of the study according to several authors [29, 30]. As a direct consequence, all the values recorded in both groups resulted to be extremely decreased at the second time point, after 1 month. However, the data collection at the third time point 3 months after the baseline made possible to analyze the new plaque formation trend in both groups and verify the different devices’ efficacy on both teeth and implant. The evolution observed over time can be related also to the presence of peri-implant and periodontal pockets. Despite that the average values of PPD were lower than 3 mm, patients presenting deeper pockets were included, which might represent a limit of the study. The prophylaxis performed at the beginning of the study, in fact, could not remove adequately the plaque present in the deepest area of these pockets. This prevented the achievement of a “level 0” of PI and BoP and, at the same time, promoted a faster re-colonization. During this time, patients also improved their skills with the electric toothbrush, which have also might influence their motivation. These factors could explain the reduction of PI observed in the second part of the study on the electric toothbrush groups and, sequentially, of the BoP as inflammatory index caused by the presence of plaque itself. The efficacy of the electric toothbrush can be related to the easiness of use and the complexity of artificial movement (rotating-oscillatory), which has been demonstrated to be more effective in plaque removal with respect to the manual toothbrush as reported by many authors [14, 25, 27]. Many authors observed a 0.3-mm reduction of probing depth after at least 12-month observation period in the patients using the electric toothbrush [26, 28]. Despite in the present study it was observed only 0.15 mm of mean probing reduction for dental implants, our observation was limited only to a 3-month period. This trend could be comparable to a 0.3-mm reduction in 12 months, as observed in the previous studies. However, a similar trend was also detected in the control group so the electric toothbrush cannot be directly related to the PPD reduction.

At the end of the present study, electric toothbrush groups showed plaque and bleeding values lower (PI and BoP on teeth) or at least without significative differences (BoP on implants) than the control group. These data may suggest how the use of electric toothbrush, associated to the dedicate heads, can be an effective method for plaque and bleeding reduction.

## Conclusion

The oscillating-rotating toothbrush can be used for the plaque and bleeding control around both natural teeth and dental implants. It has also been shown how the toothbrush head designed for dental implant can be effective in plaque removing of the peri-implant tissues.

## Abbreviations

BoP: Bleeding on probing
PI: Plaque index
PPD: Pocket probing depth
